# ^131^I-metaiodobenzylguanidine (^131^I-MIBG) therapy for residual neuroblastoma: a mono-institutional experience with 43 patients

**DOI:** 10.1038/sj.bjc.6694223

**Published:** 1999-12

**Authors:** A Garaventa, O Bellagamba, M S Lo Piccolo, C Milanaccio, E Lanino, L Bertolazzi, G P Villavecchia, M Cabria, G Scopinaro, F Claudiani, B De Bernardi

**Affiliations:** Department of Haematology–Oncology, Giannina Gaslini Children’s Hospital, Genova-16147, Italy; Service of Nuclear Medicine, Galliera Hospital, Genova-16123, Italy

**Keywords:** neuroblastoma, radiometabolic therapy, ^131^I-metaiodobenzylguanidine

## Abstract

Incomplete response to therapy may compromise the outcome of children with advanced neuroblastoma. In an attempt to improve tumour response we incorporated ^131^I-metaiodobenzylguanidine (^131^I-MIBG) in the treatment regimens of selected stage 3 and stage 4 patients. Between 1986 and 1997, 43 neuroblastoma patients older than 1 year at diagnosis, 13 with stage 3 (group A) and 30 with stage 4 disease (group B) who had completed the first-line protocol without achieving complete response entered in this study. ^131^I-MIBG dose/course ranged from 2.5 to 5.5 Gbq (median, 3.7). The number of courses ranged from 1 to 5 (median 3) depending on the tumour response and toxicity. The most common acute side-effect was thrombocytopenia. Later side-effects included severe interstitial pneumonia in one patient, acute myeloid leukaemia in two, reduced thyroid reserve in 21. Complete response was documented in one stage 4 patient, partial response in 12 (two stage 3, 10 stage 4), mixed or no response in 25 (ten stage 3, 15 stage 4) and disease progression in five (one stage 3, four stage 4) Twenty-four patients (12/13 stage 3, 12/30 stage 4) are alive at 22–153 months (median, 59) from diagnosis. ^131^I-MIBG therapy may increase the cure rate of stage 3 and improve the response of stage 4 neuroblastoma patients with residual disease after first-line therapy. A larger number of patients should be treated to confirm these results but logistic problems hamper prospective and coordinated studies. Long-term toxicity can be severe. © 1999 Cancer Research Campaign


*British Journal of Cancer* (1999) **81**(8), 1378–1384
© 1999 Cancer Research Campaign
Article no. bjoc.1999.0855

### $^{131}$I-metaiodobenzylguanidine ($^{131}$I-MIBG) therapy for residual neuroblastoma: a mono-institutional experience with 43 patients

**A Garaventa[1], O Bellagamba[1], M S Lo Piccolo[1], C Milanaccio[1], E Lanino[1], L Bertolazzi[2], GP Villavecchia[2], M Cabria[2], G Scopinaro[2], F Claudiani[2] and B De Bernardi[1]**

[1]Department of Haematology–Oncology, Giannina Gaslini Children's Hospital, 16147-Genova, Italy; [2]Service of Nuclear Medicine, Galliera Hospital, 16123-Genova, Italy

**Summary** Incomplete response to therapy may compromise the outcome of children with advanced neuroblastoma. In an attempt to improve tumour response we incorporated $^{131}$I-metaiodobenzylguanidine ($^{131}$I-MIBG) in the treatment regimens of selected stage 3 and stage 4 patients. Between 1986 and 1997, 43 neuroblastoma patients older than 1 year at diagnosis, 13 with stage 3 (group A) and 30 with stage 4 disease (group B) who had completed the first-line protocol without achieving complete response entered in this study. $^{131}$I-MIBG dose/course ranged from 2.5 to 5.5 Gbq (median, 3.7). The number of courses ranged from 1 to 5 (median 3) depending on the tumour response and toxicity. The most common acute side-effect was thrombocytopenia. Later side-effects included severe interstitial pneumonia in one patient, acute myeloid leukaemia in two, reduced thyroid reserve in 21. Complete response was documented in one stage 4 patient, partial response in 12 (two stage 3, 10 stage 4), mixed or no response in 25 (ten stage 3, 15 stage 4) and disease progression in five (one stage 3, four stage 4) Twenty-four patients (12/13 stage 3, 12/30 stage 4) are alive at 22–153 months (median, 59) from diagnosis. $^{131}$I-MIBG therapy may increase the cure rate of stage 3 and improve the response of stage 4 neuroblastoma patients with residual disease after first-line therapy. A larger number of patients should be treated to confirm these results but logistic problems hamper prospective and coordinated studies. Long-term toxicity can be severe. © 1999 Cancer Research Campaign

**Keywords**: neuroblastoma; radiometabolic therapy; $^{131}$I-metaiodobenzylguanidine

One of the major goals of cancer treatment is to develop therapies affecting cancer cells while causing little or no damage to the normal counterparts. In this perspective numerous attempts have been made to bind anti-tumour compounds or radioactive isotopes to molecules specifically taken up by tumour cells. One example of these molecules is benzylguanidine, a structural analogue of noradrenaline, which is selectively taken up by cells of neural crest origin including tumours such as pheochromocytoma and neuroblastoma (Jaques et al, 1987; Montaldo et al, 1991). Linking radioactive iodine to benzylguanidine has led to the synthesis of $^{123}$I- and $^{131}$I-metaiodobenzylguanidine (*I-MIBG), which at low doses have become an important tool for both diagnosis and follow-up of these tumours (Buck et al, 1985; Geatti et al, 1985). Moreover, given at higher doses $^{131}$I-MIBG has proven to be active against these tumours, especially neuroblastoma (Schwabe et al, 1987; Klingebiel et al, 1989). Most clinical studies have been carried out on patients in advanced stages of the neoplasia (Italian MIBG Workshop, 1987; Klingebiel et al, 1989; Lashford et al, 1992), although recently $^{131}$I-MIBG has been administered to poor-risk patients as up-front therapy in an attempt to improve their outcome (Mastrangelo et al, 1993, 1998; Weber et al, 1996).

*Received 22 September 1998*
*Revised 4 June 1999*
*Accepted 8 June 1999*

*Correspondence to:* B De Bernardi

Despite all this information the precise role of $^{131}$I-MIBG in the overall therapeutic strategy of neuroblastoma is far from being defined. In particular, it is still unclear whether $^{131}$I-MIBG might improve the tumour response of patients who did not achieve complete remission with conventional therapy and are therefore predisposed to disease progression and death. Since 1986 it has been the policy of our institute to use $^{131}$I-MIBG as therapy for stage 3 and 4 neuroblastoma patients with MIBG-positive residual disease after front-line protocol. We have thus accumulated considerable experience in this area.

The results of this experience are hereby reported. They suggest that $^{131}$I-MIBG may provide additional therapeutic benefits for some of these patients, although related toxicity may occasionally be severe.

### MATERIALS AND METHODS

### Patients

Patients were registered in this study between March 1986 and December 1996. They were diagnosed with inoperable or disseminated neuroblastoma (INSS stage 3 and stage 4 respectively) (Brodeur et al, 1993) in ages ranging from 1 to 15 years. Diagnosis of neuroblastoma and evaluation of disease extent was carried out according to standard clinical and pathologic criteria of the Italian Cooperative Group for Neuroblastoma (ICGNB) (De Bernardi et al, 1992). Treatment was given according to ICGNB protocols (De Bernardi et al, 1992). In order to be eligible for this study

**Table 1** Stage 3 patients (group A)

| Case no. | Sex, age (yrs) | Primary tumour site | NMYC copies | Time (months) from diagnosis to $^{131}$I-MIBG therapy | Tumour lesion at $^{131}$I-MIBG therapy | | $^{131}$I-MIBG therapy courses | Tumour response after $^{131}$I-MIBG therapy | Clinical course and outcome |
|---|---|---|---|---|---|---|---|---|---|
| | | | | | Primary tumour | VMA–HVA urinary | | | |
| 1 | F, 2 | Thorax | 1 | 7 | Yes | E | 2 | NR | Alive 131 months, SD |
| 2 | M, 3 | Thorax | 1 | 6 | Yes | E | 4 | PR | Alive 107 months, CR |
| 3 | F, 2 | RPG | 1 | 5 | Yes | E | 3 | NR | Alive 98 months, SD |
| 4 | M, 12 | Adrenal | 1 | 14 | Yes | E | 2 | NR | Alive 89 months, SD |
| 5 | F, 1 | RPG | ND | 1 | Yes | E | 4 | NR | Alive 78 months, CR |
| 6 | F, 2 | RPG | 32 | 7 | Yes | E | 3 | PR | Alive 79 months, CR |
| 7 | M, 3 | RPG | 1 | 7 | Yes | E | 4 | NR | Alive 77 months, SD |
| 8 | F, 3 | Thorax | 1 | 8 | Yes | E | 3 | NR | Alive 71 months, SD |
| 9 | M, 1 | RPG | 1 | 5 | Yes | E | 4 | MR | Alive 56 months, SD |
| 10 | F, 1 | Adrenal | 1 | 5 | Yes | E | 4 | NR | Alive 56 months, SD |
| 11 | F, 4 | RPG | 14 | 8 | Yes | E | 3 | PD | Dead 17 months, PD |
| 12 | M, 2 | Adrenal | 1 | 6 | Yes | E | 1 | NR | Alive 47 months, CR |
| 13 | M, 1 | RPG | 1 | 8 | Yes | E | 2 | NR | Alive 34 months, SD |

RPG, retroperitoneal ganglia; B, bone; (s), single lesion; (m), multiple lesions; BM, bone marrow; LN, distant lymph node; CR, complete remission; PR, partial remission; MR, minor response; NR, no response; SD, stable disease; PD, progressive disease; N normal, E elevated, ND, not done. [a]Acute myeloid leukaemia presently in remission. [b]Acute myeloid leukaemia under treatment.

patients had to have either (a) stage 3 disease with residual tumour positive at the $^{123}$I- or $^{131}$I-MIBG scintigraphy at end of first-line therapy (group A), or (b) stage 4 disease partially responsive to first-line therapy with residual tumour at the level of primary tumour and/or skeleton (no more than four lesions) and/or bone marrow (only if infiltration was of minimal entity) with at least one lesion clearly uptaking $^{123}$I- or $^{131}$I-MIBG (group B). The cohort of 43 patients in this study (13 stage 3 and 30 stage 4) represents all the patients with these characteristics treated in our institution. This accounts for approximately one-third of the patients enrolled in the Italian protocols between 1985 and 1996 who reached partial response to first-line therapy (28 out of 76 stage 3 and 102 out of 281 stage 4 patients). In the same period we diagnosed and treated 36 stage 3 and 112 stage 4 patients aged more than 1 year.

Further eligibility criteria included recovery of haematopoiesis from previous chemotherapy, and normal renal, hepatic and thyroid functions. Parents or guardian were informed of the experimental nature of this treatment and requested to give written consent. The study was approved by the Ethical Committee of the Giannina Gaslini Children’s Hospital.

### Evaluation before $^{131}$I-MIBG therapy

Pretreatment evaluation included physical examination, complete blood cell count with platelet and differential white cell count, blood tests showing normal thyroid, kidney and liver functions, imaging studies of the primary tumour with computerized tomography and/or magnetic resonance imaging and $^{123}$I- or $^{131}$I-MIBG scintigraphy, measurement of tumour markers (vanillylmandelic [VMA] and homovanillic [HVA] acids in urine; ferritin, neuron-specific enolase and lactate dehydrogenase in serum) and marrow evaluation consisting of four aspirations and two trephine biopsies.

Disease evaluation to assess tumour response was performed between 3 and 6 weeks after each $^{131}$I-MIBG course.

### Evaluation of tumour response

Tumour response was defined as follows (Brodeur et al, 1993): complete response (CR), disappearance of primary tumour and of all metastatic lesions with normalization of urine catecholamines; very good partial response (VGPR), > 90% volume reduction of the primary tumour with clearing of all measurable metastatic lesions with the exception of residual changes at skeletal scintigraphy, normalization of urine catecholamines; partial response (PR), > 50% volume reduction of the primary tumour and of all measurable metastatic lesions, residual marrow infiltration in only one site; mixed response (MR), > 50% reduction of any measurable lesion (primary or metastases) with < 50% reduction in any other and < 25% increase in any existing lesion; no response (NR), < 50% reduction but < 25% increase in any existing lesion; progressive disease (PD), increase > 25% of any measurable lesion or appearance of a new lesion(s).

The isolated decrease of $^{131}$I-MIBG uptake was not considered evidence of tumour response. Response was evaluated 4–8 weeks following each $^{131}$I-MIBG course. The best response achieved is reported in the Results section of the article and in the Tables. Some patients underwent further improvement several months after completion of $^{131}$I-MIBG therapy and the best status achieved is reported in the outcome.

### $^{131}$I-MIBG therapy

The administered doses of $^{131}$I-MIBG ranged from 2.5 to 5.5 GBq according to body weight: 2.5–3.7 GBq for patients weighing less than 15 kg; 3.7–4.7 GBq for patients weighing 15–20 kg; 5.5 GBq for those weighing more than 20 kg. Specific activity ranged from 1.1 to 2.8 GBq mg$^{-1}$ (median 1.6). Doses were determined attempting to give at least 2000 cGy to the tumour and possibly less then 200 cGy to the whole body (Beirewaltes, 1987).

Thyroid gland uptake of radioiodine was blocked by oral administration of iodine (2–3 mg kg$^{-1}$ day$^{-1}$ of iodine as Lugol’s solution) given for 5 days before and 8 days after $^{131}$I-MIBG

© 1999 Cancer Research Campaign

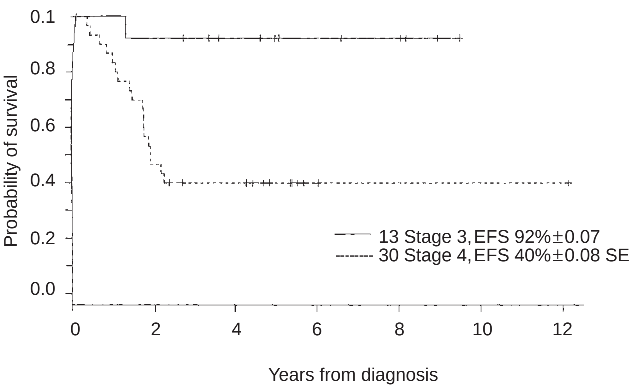

**Figure 1** EFS in stage 3 and four patients who received I[131]-MIBG therapy for residual neuroblastoma infusion. During hospitalization patients were kept in single rooms. Close relatives actively participated in nursing care and were provided with film dosimetry. Once discharged patients were checked weekly for haematological indices and at longer intervals for other organic functions.

Toxic effects attributable to $^{131}$I-MIBG therapy were evaluated according to WHO criteria (WHO, 1979). The duration of $^{131}$I-MIBG treatment depended on tumour response and toxicity. In case of either disease improvement or stability after the first $^{131}$I-MIBG course, additional courses up to a maximum of 5 with an interval of 4–6 weeks between courses were administered unless evidence of progressive disease was documented or excessive myelotoxicity had occurred.

### RESULTS

Group A consisted of 13 stage 3 disease patients who had had partial tumour response to first-line regimen. Before $^{131}$I-MIBG therapy 11 patients underwent second-look surgery consisting of partial tumour resection in four and of a biopsy only in seven with histological evidence of viable tumour cells in all surgical specimens. At the time of entry into this study all patients but one had abnormal urinary catecholamine excretion. All showed pathological scintigraphic *I-MIBG uptake at the site of the primary.

Main patients' data including characteristics at diagnosis, interval from diagnosis to $^{131}$I-MIBG therapy, number of courses of $^{131}$I-MIBG administered, tumour response to $^{131}$I-MIBG and outcome are listed in Table 1. In all but one patient NMYC gene

**Table 2** Stage 4 patients (group B)

| Case no. | Sex, age (years) | Primary tumour site | NMYC copies | Time (months) from diagnosis to $^{131}$I-MIBG therapy | Tumour lesions at $^{131}$ I-MIBG therapy | | | $^{131}$I-MIBG therapy courses therapy | Tumour response after $^{131}$I-MIBG | Clinical course and outcome |
|---|---|---|---|---|---|---|---|---|---|---|
| | | | | | Primary tumour | Metastases | VMA–HVA urinary | | | |
| 1 | F, 3 | RPG | ND | 25 | Yes | No | E | 4 | PR | Alive 153 months, CR[a] |
| 2 | M, 4 | RPG | 1 | 10 | Yes | No | N | 2 | NR | Dead 21 months, PD |
| 3 | M, 2 | Adrenal | ND | 11 | Yes | No | E | 3 | NR | Death 22 months, PD |
| 4 | M, 2 | RPG | 32 | 7 | Yes | No | N | 3 | PR | Alive 67 months, CR |
| 5 | F, 1 | Adrenal | 9 | 7 | Yes | No | N | 1 | PR | Alive 65 months, CR |
| 6 | F, 17 | RPG | 1 | 21 | Yes | No | E | 2 | NR | Alive 57 months, SD |
| 7 | F, 3 | RPG | 1 | 13 | Yes | No | N | 2 | PR | Alive 47 months, SD |
| 8 | M, 1 | RPG | 1 | 12 | Yes | No | N | 2 | NR | Alive 36 months, SD |
| 9 | F, 2 | Adrenal | 1 | 9 | Yes | No | E | 2 | NR | Dead 21 months, PD |
| 10 | M, 14 | RPG | 1 | 18 | Yes | No | N | 2 | NR | Alive 30 months, SD |
| 11 | F, 2 | RPG | 1 | 5 | Yes | O(m), MO | N | 3 | NR | Dead 10 months, PD |
| 12 | M, 3 | Adrenal | 20 | 6 | Yes | O(m), MO | E | 5 | NR | Dead 14 months, PD |
| 13 | M, 4 | Adrenal | ND | 16 | Yes | O(m) | E | 1 | PD | Dead 19 months, PD |
| 14 | M, 2 | Adrenal | ND | 9 | Yes | LN | E | 2 | PD | Dead 12 months, PD |
| 15 | M, 6 | RPG | ND | 14 | Yes | MO | E | 2 | NR | Dead 20 months, PD |
| 16 | F, 1 | RPG | ND | 13 | Yes | O(m), MO | E | 3 | PR | Dead 19 months, PD |
| 17 | F, 4 | RPG | 1 | 11 | Yes | O(m), MO | E | 3 | PR | Alive 39 months, SD[b] |
| 18 | M, 4 | RPG | 1 | 12 | Yes | LN | E | 3 | NR | Dead 20 months, PD |
| 19 | M, 16 | RPG | 1 | 12 | Yes | LN | E | 2 | NR | Alive 48 months, SD |
| 20 | M, 11 | RPG | 14 | 9 | Yes | O(m) | N | 2 | NR | Dead 13 months, PD |
| 21 | M, 1 | RPG | 3 | 10 | Yes | LN | N | 3 | NR | Toxic death, 13 months |
| 22 | M, 2 | RPG | 20 | 10 | Yes | MO | E | 3 | PR | Dead 14 months, PD |
| 23 | M, 4 | Adrenal | 1 | 12 | Yes | B(m), BM | N | 3 | PR | Dead 16 months, PD |
| 24 | M, 5 | Adrenal | 1 | 17 | Yes | B(m), BM | N | 1 | PD | Dead 24 months, PD |
| 25 | F, 4 | Adrenal | ND | 14 | Yes | B(m), BM | E | 2 | NR | Alive 24 months, PD |
| 26 | M, 4 | RPG | 1 | 10 | Yes | B(s), BM | N | 1 | NR | Alive 22 months, SD |
| 27 | M, 2 | Adrenal | 1 | 8 | No | BM | N | 4 | PD | Dead 9 months, PD |
| 28 | F, 1 | Adrenal | 1 | 12 | No | B(s) | E | 3 | PR | Alive 60 months, CR |
| 29 | F, 3 | Adrenal | 1 | 17 | No | B(m) | N | 1 | CR | Alive 68 months, CR |
| 30 | M, 2 | Adrenal | 3 | 21 | No | BM | E | 2 | PR | Alive 45 months, PD |

RPG, retroperitoneal ganglia; B, bone; (s), single lesion; (m), multiple lesions; BM, bone marrow; LN, distant lymph node; CR, complete remission; PR, partial remission; MR, minor response; NR, no response; SD, stable disease; PD, progressive disease; N normal, E elevated, ND, not done. [a]Acute myeloid leukaemia presently in remission. [b]Acute myeloid leukaemia under treatment.

 *© 1999 Cancer Research Campaign*

was studied, and in two patients an abnormal copy number was present.

One patient received one $^{131}$I-MIBG course, three patients received two courses, four received three courses and five received four courses. The interval between courses ranged from 4 to 8 weeks (median, 7). Two patients had partial and one had mixed response (< 25% reduction of tumour volume with return to normal of catecholamines). The disease remained stable in nine patients and progressed in one alone.

No further antitumour therapy was administered and four patients became progressively disease-free including normal *I-MIBG scan despite some 'irregularity' detected by imaging studies at level of primary tumour and interpreted as possible scar tissue. They are now alive at 47–107 months (median, 78) from diagnosis (case nos 2, 5, 6, 12). Eight patients are alive with stable disease (residual tumour persisting at imaging and MIBG scan) at 24–131 months (median, 74). Four of them underwent biopsy of the residual tumour with histological findings of ganglioneuroma in two (case nos 3, 7) and of ganglioneuroblastoma in the other two (case nos 4, 9). The only case (no. 11) who developed PD after $^{131}$I-MIBG therapy died 9 months later. He was one of the two patients with an amplified NMYC gene. The 5-year event-free survival (EFS) of this group is 92% (± 0.07) with follow-up of 34–131 months (median, 78) (Figure 1).

Group B consisted of 30 patients with stage 4 disease who achieved partial response with first-line therapy. Twenty-one received $^{131}$I-MIBG therapy within 2 months from completion of their chemotherapy protocol ending in a course of myeloablative therapy, while in the remaining nine patients $^{131}$I-MIBG therapy was not preceded by myeloablative therapy due to (a) inoperable primary tumour (five patients), (b) bone marrow infiltration by tumour preventing autologous stem cell harvest (two patients), and (c) lack of adequate harvest (two patients). Scintigraphy performed before $^{131}$I-MIBG therapy was positive at one site in 11 patients (at level of primary in ten, a unique bone lesion in one), two sites in five, three or more sites in 14.

Main patient data are summarized in Table 2. Five patients received one $^{131}$I-MIBG course of therapy, 13 received two courses, nine received three courses, two received four courses and one received five courses. Intervals between courses ranged from 4 to 16 weeks (median, 7).

One patient (case no. 29) achieved CR (clearing of two bone lesions) and is presently alive disease-free 50 months later. Eleven patients had PR involving the primary tumour (the only tumour lesion) in four cases (nos 1, 4, 5, 7), a single bone lesion in one case (case no. 28), bone marrow in one (case no. 30), bone marrow plus primary in one (case no. 22), and bone plus bone marrow and primary in three (case nos 16, 17, 23). Eight of them are alive at 36–153 months (median, 53) among whom four are in CR (case nos 1, 4, 5, 28) including case no. 1 who developed myeloid leukaemia and is presently in CR after allogeneic bone marrow graft, two cases who had no further disease change (case nos 8, 17) including case no. 17 who developed myeloid leukaemia and is still alive after 14 months and one (case no. 30) who is alive with PD. Fourteen patients did not respond. Among them one is alive with PD at 24 months (case no. 25) and five are alive with stable disease (case nos 6, 8, 10, 19, 26) at 22–88 months (median, 42) from diagnosis, while the remaining nine patients died, among whom seven of disease at 13–21 months (median, 20) and two of toxicity.

**Table 3** Haematological toxicity after $^{131}$I-MIBG therapy

| Course | | I | II | III | >IV | Total |
|---|---|---|---|---|---|---|
| Stage 3: evaluable course | | 12 | 11 | 7 | 2 | 32 |
| Leukopenia: | grade 3 | – | 1 | – | – | 1 |
| | 4 | – | – | – | – | – |
| Thrombocytopenia: | grade 3 | – | 3 | – | – | 3 |
| | 4 | – | 1 | 1 | – | 2 |
| Stage 4: evaluable courses | | 25 | 21 | 9 | 3 | 58 |
| Leukopenia: | grade 3 | 2 | 5 | 1 | – | 8 |
| | 4 | – | – | – | – | – |
| Thrombocytopenia: | grade 3 | 9 | 3 | 1 | – | 13 |
| | 4 | 8 | 7 | 3 | 1 | 19 |

The last four patients (case nos 13, 14, 24, 27) experienced early PD and died within a few months. The 5-year EFS of this group is 40% (± 0.08) (Figure 1).

Patients having a single tumour lesion positive at *I-MIBG scan had better chance of survival with eight out of 11 surviving, four of whom in CR. Of the four patients with two positive lesions, two are presently alive with non-progressive disease and one is alive in CR. Of the 14 with three or more *I-MIBG-positive lesions, only three are currently alive among whom two with stable disease and one with PD.

Bone marrow infiltration at time of $^{131}$I-MIBG therapy was prognostically unfavourable, since only three of 11 such patients are presently alive, one with stable disease and two with PD.

Of the 18 patients with normal NMYC copy number, 11 are alive; out of four patients with amplified NMYC only one survives, and two out of seven patients not evaluated for this gene survive.

The majority of patients who responded to $^{131}$I-MIBG therapy and survive are those who received $^{131}$I-MIBG after myeloablative therapy. In fact, out of 21 such patients nine responded (one CR, eight PR), nine had no disease change (one died of toxicity and eight progressed) and three developed progressive disease. On the contrary, in the group of nine patients who were not eligible for such a therapy only one responded, although a total of four are alive with stable disease.

### Toxicity

### *Acute toxicity*

No noticeable reaction was registered during or shortly after the administration of $^{131}$I-MIBG courses.

### *Haematological toxicity (Table 3)*

Among the group A patients the main toxicity of this type attributable to $^{131}$I-MIBG was thrombocytopenia. However, out of 32 evaluable courses only two patients had grade 4 thrombocytopenia and three others had grade 3 toxicity which did not worsen with subsequent courses. Six patients, for a total of 24 courses, manifested no haematological toxicity.

In group B patients 58/72 courses were evaluable. Only one patient had no marrow toxicity. Grade 4 thrombocytopenia occurred after 19 course* grade 3 leukopenia occurred after 18 courses. None of the 21 patients who received therapeutic $^{131}$I-MIBG after myeloablative chemotherapy and autologous stem cell rescue had grade 4 haematological toxicity, although 12 developed grade 3 and seven grade 2 thrombocytopenia, which was often long-lasting (2–7 months; median 5).

© 1999 Cancer Research Campaign

Both thrombocytopenia and leukopenia occurred in most cases 3–5 weeks following administration of $^{131}$I-MIBG therapy. Lymphocytopenia below $1 \times 10^9$ L$^{-1}$ was found only after nine of 50 evaluable courses and appeared earlier (7–10 days after $^{131}$I-MIBG infusion) than other haematological toxicities.

### Other toxicities

One stage 4 disease patient (case no. 21) treated with megatherapy and autologous bone marrow rescue, followed by three courses of $^{131}$I-MIBG therapy, developed interstitial pneumopathy 1 month after the last course and died. One stage 4 patient (case no. 17) developed acute myeloid leukaemia (FAB M2) 1 year after the last (third) $^{131}$I-MIBG course. She had previously received carboplatin for a total dose of 4.4 g m$^{-2}$, cyclophosphamide for 14.4 g m$^{-2}$, etoposide for 1.5 mg m$^{-2}$ and melphalan for 140 mg m$^{-2}$, plus 27 Gy radiation therapy to the primary tumour site. Another stage 4 disease patient (case no. 1) who had received 1.8 g m$^{-2}$ of peptichemio, 600 mg m$^{-2}$ of cisplatin and only one course of $^{131}$I-MIBG, developed myeloid leukaemia 6 years later, which was successfully treated by an unrelated marrow graft. This patient is presently alive disease-free 12 years after the initial diagnosis.

Twenty-one patients showed compensated hypothyroidism (high thyroid-stimulating hormone plasma level with normal $T_3$ and $T_4$ values). Thyroid abnormalities appeared between 1 and 44 months (median, 12) after the first dose of $^{131}$I-MIBG. Subsequently, 15 patients developed overt hypothyroidism requiring replacement treatment with L-thyroxine at 2–56 months (median, 19) after initiation of $^{131}$I-MIBG therapy. Thyroid function remained normal in 19 patients having at least 1 year of follow-up (17–98 months, median, 45).

### DISCUSSION

Long-term survival of children with inoperable or disseminated neuroblastoma diagnosed after the age of 1 year remains largely unsatisfactory mainly because current treatment commonly fails to eradicate the disease (Garaventa et al, 1993; Haase et al, 1995; Philip et al, 1997). Thus, until innovative and more effective therapies are developed the paediatric oncologist dealing with refractory or partially responsive neuroblastoma must struggle to optimize the therapeutic tools at his disposal (Castleberry et al, 1991; Gaze et al, 1995; Leavey et al, 1997).

When radioiodinated benzylguanidine became available for the diagnosis of neural crest-derived tumours the possibility to use it at much higher doses for therapeutic purposes brought about a great deal of interest. Both $^{125}$I- and $^{131}$I-labelled MIBG seemed suitable due to their different physical characteristics. The former emits short-range energy making it theoretically suitable to destroy small tumour aggregates (Buck et al, 1985), while the long-range radiation emitted of $^{131}$I could be more effective to induce damage of larger tumour lesions (Weber et al, 1996). However, since residual neuroblastoma is usually not homogeneous, $^{125}$I short-range radioactivity might miss part of the tumour and was consequently abandoned with few exceptions (Sisson et al, 1996) and the therapeutic use of radioactive MIBG has been limited to $^{131}$I-MIBG.

Initial phase I–II studies with $^{131}$I-MIBG therapy, dating back to the late 1980s, showed good tolerance and demonstrated objective tumour responses in 20–60% of patients failing to respond to modern therapies (Italian MIBG Workshop, 1987; Schwabe et al, 1987; Klingebiel et al, 1989; Lashford et al, 1992). In addition, significant pain relief was noticed even in patients who did not exhibit objective responses (Klingebiel et al, 1989). Since the anti-tumour effect is dose-dependent with a maximum tolerated dose of 12 mCi kg$^{-1}$, autologous stem cell rescue has been used to allow dose escalation up to 18 mCi kg$^{-1}$ (Matthay et al, 1998). Lastly, attempts are being made to evaluate whether $^{131}$I-MIBG given as front-line therapy may improve the outcome of these patients without causing excessive toxicity (Van Hasselt et al, 1996; Mastrangelo et al, 1998). No prospective study has been carried out so far to the true potential value of MIBG therapy and to identify the group of patients who may profit best from this treatment modality. The difficulties in design such studies include the rarity of neuroblastoma, the fact that many centres do not possess the facilities to deliver the treatment to young patients, the high cost of the drug and, not least, the psychological burden of keeping patient in a radiation protected environment with limited parental contact extended for several days.

In a previous study we reported on 31 patients treated after relapse or progression: the rate and degree of responses were lower in the presence of bulky disease, a high number of MIBG-positive lesions, long duration of previous therapy and overt bone marrow infiltration (Garaventa et al, 1991).

In the present study we administered $^{131}$I-MIBG as therapy to children with high-risk neuroblastoma who responded to first-line therapy without achieving CR, provided that the residual primary tumour and/or metastase(s) were clearly uptaking MIBG. Our objective was to increase knowledge on toxicity of repeated cycles and the possibly to improve the outcome of these children. Our results indicate that stage 3 disease patients did benefit from this treatment. Despite the fact that only two out of 13 of them had a greater than 50% decrease of the residual primary tumour at short-term evaluation after completion of $^{131}$I-MIBG therapy, two others later experienced a progressive decrease in tumour size until its disappearance without receiving further anti-tumour therapy. The disease has remained stable for a median of 75 months in eight other patients. Four of these eight patients eventually underwent biopsy of the residual tumour, which turned out to be ganglioneuroma in two cases and ganglioneuroblastoma, with marked evidence of differentiation in the other two. The disease progressed in only one patient after $^{131}$I-MIBG therapy. It is of interest that this patient had abnormal MYCN gene copy number, although a similar patient is a long survivor. Presently, 12 out of 13 patients are alive either disease-free or with stable disease with an observation period of several years in most cases (Figure 1).

Since several authors have reported that incomplete tumour excision in stage 3 disease is associated with a worse survival rate (Garaventa et al, 1993; Haase et al, 1995; Powis et al, 1996), we believe that our results should be considered worthy of interest despite the fact that only one of our long-term survivors had amplification of MYCN gene.

The possible benefit of $^{131}$I-MIBG therapy for stage 4 disease patients is more difficult to prove in this study. Among 30 such patients only one achieved a CR and survives. However, ten other patients experienced a PR and six of them are alive. No tumour change was seen in 15 patients, six of whom are alive despite the absence of additional treatment. Four patients developed early progression and died. Tumour response and outcome correlated with the extent of the disease at the time of $^{131}$I-MIBG therapy. In fact, of 11 patients with a single positive lesion at $^{123}$I-MIBG scintigraphy eight survive with stable disease, while of 19 with two or more positive lesions only five survive. This last group includes 12 patients with bone marrow involvement of whom only two survive without tumour progression.

 © 1999 Cancer Research Campaign

Twenty-one of the 30 stage 4 disease patients received therapeutic $^{131}$I-MIBG within 3 months from myeloablative therapy. Although we expected severe and long-lasting haematological toxicity in these patients, they tolerated the radioiodinated treatment surprisingly well. In particular no severe haemorrhagic episodes were recorded, though three patients required several platelet transfusions.

However, important non-haematological toxicity which was not encountered in stage 3 disease, did occur in stage 4, possibly due to the overall greater amount of chemotherapy usually given to these patients as well as occasional irradiation to large proportions of the bone marrow and skeleton. One example was the early occurrence of fatal interstitial pneumonia that one patient developed within 2 months after the last $^{131}$I-MIBG therapy course. The precocity of this complication suggests that the elevated concentration of the radioactive compound in the pulmonary bloodstream during $^{131}$I-MIBG therapy may aggravate a pre-existing immune deficiency favouring the growth of opportunistic micro-organisms. This hypothesis is confirmed by the increased risk of pneumopathy reported in lymphoma patients treated with $^{131}$I-charged monoclonal antibodies (Press et al, 1995). According to these authors, after bone marrow, the organs which are most likely to develop toxicity from radioiodinated therapy are the lungs. This should therefore be taken into consideration when planning doses and duration of this type of treatment. Even more concern raises the early occurrence of leukaemia in two out of 30 stage 4 disease patients since it could represent the result of a combined chemo-radiotherapeutic effect on bone marrow precursor cells. A similar case has recently been reported in a series of 30 children enrolled in a therapeutic $^{131}$I-MIBG dose-finding study (Matthay et al, 1998). If the risk of secondary leukaemia in neuroblastoma patients subjected to $^{131}$I-MIBG therapy proved true, then the limits of this treatment would have to be more clearly defined. Subclinical hypothyroidism was frequent in our series. Statistical analysis to determine the role of factors such as age, numbers and doses of $^{131}$I-MIBG courses could not be performed due to the small number of cases. Compliance to thyroid blockade is a critical point, however, although greater attention will be paid to minimize this complication in the future and a close follow-up of thyroid status is necessary. Few patients required chronic substitutive therapy and no significant impact on the quality of life was reported.

In conclusion, our data suggest that $^{131}$I-MIBG therapy may provide additional anti-tumour effects in stage 3 neuroblastoma with residual disease after first-line therapy and this may translate into an overall improved outcome. This favourable result has been reached with the only side-effects being tolerable haematological and thyroid toxicity. In our opinion, the fact that 12 out of 13 such patients are alive could justify a prospective study of $^{131}$I-MIBG therapy in this particular subset of patients. $^{131}$I-MIBG exhibited antitumour activity in approximately one-third of stage 4 patients. Interestingly enough, the haematological toxicity of this treatment was of moderate entity even for patients who had previously received myeloablative therapy. Overall, our results suggest that the addition of $^{131}$I-MIBG at therapeutic dosages to the conventional regimens for high-risk neuroblastoma may implement tumour response in some patients and this might translate in better chance of cure for some of them. Controlled clinical trials should be considered to define the true potential of this new therapeutic tool. However, since trials aiming to respond such an important question would imply the long lasting commitment of highly specialized centres and a potential severe toxicity the cost– benefit ratio of such a study deserves to be carefully eveluated.

### ACKNOWLEDGEMENTS

The authors acknowledge the secretarial assistance of Ms Milena Dondero. Supported in part by grant no. 95.00494.PF39, Consiglio Nazionale delle Ricerche, Roma; and a special fund of the Associazione Italiana per la Lotta al Neuroblastoma, Genova, Italy

### REFERENCES

Beirewaltes WH (1987) Treatment of neuroblastoma with $^{131}$I-MIBG: dosimetric problems and prospectives. *Med Pediatr Oncol* **15**: 188–191

Bolster A, Hilditch TE, Wheldon TE, Gaze MN and Barrett A (1995) Dosimetric considerations in $^{131}$I-MIBG therapy for neuroblastoma in children. *Br J Radiol* **68**: 481–490

Buck J, Bruchelt G and Girgert R (1985) Specific uptake of m (125) iodobenzylguanidine in the human neuroblastoma cell line SK-N-SH. *Cancer Res* **45**: 6366–6370

Brodeur GM, Pritchard J, Berthold F, Carsen NLT, Castel V, Castelberry RP, De Bernardi B, Evans AE, Favrot M, et al (1993) Revisions of the international criteria for neuroblastoma diagnosis, staging and response to treatment. *J Clin Oncol* **11**: 1466–1477

Castleberry RP, Kun LE, Shuster JJ, Altshuler G, Smith IE, Nitschke R, Wharam M, McWilliams N, Joshi V and Hayes FA (1991) Radiotherapy improves the outlook for patients older than 1 year with Pediatric Oncology Group stage C neuroblastoma. *J Clin Oncol* **19**: 789–795

De Bernardi B, Carli M, Casale F, Corciulo P, Cordero di Montezemolo L, De Laurentis C, Bagnulo S, Brisigotti M, Marchese N, Garaventa A and Felici L (1992) Standard-dose and high-dose peptichemio and cisplatin in children with disseminated poor-risk neuroblastoma: two studies by the Italian Cooperative Group for neuroblastoma. *J Clin Oncol* **10**: 1870–1878

Flower MA and Fielding SL (1996) Radiation dosimetry for $^{131}$I-MIBG therapy of neuroblastoma. *Phys Med Biol* **41**: 1933–1940

Garaventa A, Guerra P, Arrighini A, Bertolazzi L, Bestagno M, De Bernardi B, Lanino E, Villavecchia GP and Claudiani F (1991) Treatment of advanced neuroblastoma with I-131 meta-iodobenzylguanidine. *Cancer* **67**: 922

Garaventa A, De Bernardi B, Pianca C, Donfrancesco A, Cordero di Montezemolo L, Di Tullio MT, Bagnulo S, Mancini A, Carli M, Pession A, Arrighini A, Di Cataldo A, Tamaro P, Iasonni V, Taccone A, Rogers D and Boni L (1993) Localized but unresectable neuroblastoma: treatment and outcome of 145 cases. *J Clin Oncol* **9**: 1770–1779

Gaze MN, Wheldon T, O'Donoghue JA, Hilditch TE, McNee SG, Simpson E and Barrett A (1995) Multi-modality megatherapy with [$^{131}$I]metaiodobenzylguanidine, high dose melphalan and total body irradiation with bone marrow rescue: feasibility study of a new strategy for advanced neuroblastoma. *Eur J Cancer* **31**: 252–256

Geatti O, Shapiro B, Sisson JC, Hutchinson RJ, Malette S, Eyre P and Beierwaltes WH (1985) Iodine-131 metaiodobenzylguanidine scintigraphy for the location of neuroblastoma: preliminary experience in ten cases. *J Nucl Med* **26**: 736–742

Haase GM, Atkinson JB, Stram DO, Lukens JN and Matthay KK (1995) Surgical management and outcome of locoregional neuroblastoma: comparison of the Children Cancer Group and the International Staging System. *J Pediatr Surg* **30**: 289–295

Italian Society of Pediatric Oncology, Italian Society of Radiology and Nuclear Medicine, Italian Society of Biology and Nuclear Medicine and Associazione Genitori Oncologia Pediatrica (1987) International Workshop in Pediatric Oncology: the role of MIBG in therapy and diagnosis and monotoring of neuroblastoma. Rome 1986. *Med Pediatr Oncol* **17**:

Jaques S, Tobes MC and Sisson JC (1987) Sodium dependency of uptake of norepinephrine and m-iodobenzylguanidine into cultured pheochromocytoma cells: evidence for uptake on. *Cancer Res* **47**: 3920–3928

Klingebiel T, Treuner J, Ehninger G, Keller KD, Dopfer R, Feine U and Niethammer D (1989) [$^{131}$I]-metaiodobenzylguanidine in the treatment of metastatic neuroblastoma. *Cancer Chemother Pharmacol* **25**: 143–148

Lashford LS, Lewis IJ, Fielding SL, Flower MA, Meller S, Kemshead JT and Ackery D (1992) Phase I/II study of iodine $^{131}$metaiodobenzylguanidine in chemoresistant neuroblastoma: a United Kingdom Children Cancer Study Group investigation. *J Clin Oncol* **10**: 1889–1896

© 1999 Cancer Research Campaign 

Leavey PJ, Odom LF, Poole M, McNeely L, Weslie Tyson R and Haase GM (1997) Intra-operative radiation therapy in pediatric neuroblastoma. *Med Pediatr Oncol* **28**: 424–428

Mastangelo R, Lasorella A, Iavarone A, Rufini V, Troncone L, Danza F and Riccardi R (1993) Critical observation on neuroblastoma treatment with 131-I-metaiodobenzylguanidine at diagnosis. *Med Pediatr Oncol* **21**: 411–415

Mastrangelo R, Tornesello A and Mastrangelo S (1998) Role of $^{131}$metaiodobenzylguanidine in the treatment of neuroblastoma. *Med Pediatr Oncol* **31**: 22–26

Matthay KK, De Santes K and Hasegawa B (1998) Phase I dose escalation of $^{131}$I-metaiodobenzylguanidine with autologous bone marrow support in refractory neuroblastoma. *J Clin Oncol* **16**: 229–236

Montaldo PG, Lanciotti M, Casalaro A, Cornaglia-Ferraris P and Ponzoni M (1991) Accumulation of *m*-iodobenzylguanidine by neuroblastoma cells results from independent uptake and storage mechanisms. *Cancer Res* **51**: 4342–4346

Philip T, Ladenstein R, Lasset C, Hartmann O, Zucker JM, Pinkerton R, Pearson ADJ, Klingebiel T, Garaventa A, Kremens B, Bernard JL, Rosti G and Chauvin F (1997) 1070 Myeloablative megatherapy procedures followed by stem cell rescue for neuroblastoma: 17 years of European experience and conclusions. *Eur J Cancer* **33**: 2130–2135

Powis MR, Imerson JD and Homes SJK (1996) The effect of complete excision on stage III neuroblastoma: a report of the European Neuroblastoma Study Group. *J Pediatr Surg* **31**: 516–519

Press OW, Eary JF, Appelbaum FR, Martin PJ, Nelp WB, Glenn S, Fisher DR, Porter B, Matthews DC, Gooley T and Bernstein ID (1995) Phase II trial of $I^{131}$-B1 (anti-CD20) antibody therapy with autologous stem cell transplantation for relapsed B cell lymphomas. *Lancet* **346**: 336–340

Schwabe D, Sahm St, Gerein V, Happ J, Kropp-v Rabenau H, Maul F, Baum RP, Mangold K, Nitz C, Hör G and Kornhuber B (1987) 131-metaiodobenzylguanidine therapy of neuroblastoma in childhood. *Eur J Pediatr* **146**: 246–250

Sisson JC, Shapiro B, Hutchinson RJ, Shulkin BL and Zempel S (1996) Survival of patients with neuroblastoma treated with $I^{125}$-MIBG. *Am J Clin Oncol* **19**: 144–148

Van Hasselt EJ, Heij HA, De Kraker J, Vos A and Voûte PA (1996) Pretreatment with [$^{131}$I]metaiodobenzylguanidine and surgical resection of advanced neuroblastoma. *Eur J Pediatr Surg* **6**: 155–159

Weber W, Weber J and Senekowitsch-Schmidtke R (1996) Therapeutic effect of *m*-[$^{131}$I]- and *m*-[$^{125}$I] iodobenzylguanidine on neuroblastoma multicellular tumor spheroids of different sizes. *Cancer Res* **56**: 5428–5434

WHO (1979) *WHO Handbook for Reporting Results of Cancer Treatment*. WHO: Geneva

 *© 1999 Cancer Research Campaign*
